# The Utah Experience: Our Secret Sauce for Long-Term Care Nurse Residency Programs

**DOI:** 10.1093/geroni/igaa057.1723

**Published:** 2020-12-16

**Authors:** Nanci McLeskey, Joan Gallegos, Jacqueline Telonidis

**Affiliations:** 1 University of Utah, Salt Lake City, Utah, United States; 2 Comagine Health, Salt Lake City, Utah, United States

## Abstract

Nurses working in long-term care (LTC) have a paucity of geriatric knowledge and leadership skills. One method of meeting this challenge is by offering an online geriatric-focused nurse residency program (NRP) to LTC nurses. The goal of the NRP is to provide geriatric nursing competencies to prepare LTC nurses to care for complex older residents and lead staff. Construct measures include geriatric knowledge and skills, self-rated efficacy, and attitudes towards QI. To date, 46 nurses have enrolled in the program. Completers’ state they have increased knowledge and confidence; but turnover and workload issues often prevent nurses from completing the program. Thus, the current NRP with 6 nurse residents is streamlined to be completed in 4 months and with a focus on the 4 M’s, leadership and geriatric competencies and resilience. Future plans are for the NRP to be available for nurses from all long-term services and supports settings.

